# Rhythmic Neural Patterns During Empathy to Vicarious Pain: Beyond the Affective-Cognitive Empathy Dichotomy

**DOI:** 10.3389/fnhum.2021.708107

**Published:** 2021-07-07

**Authors:** Niloufar Zebarjadi, Eliyahu Adler, Annika Kluge, Iiro P. Jääskeläinen, Mikko Sams, Jonathan Levy

**Affiliations:** ^1^Department of Neuroscience and Biomedical Engineering, Aalto University, Espoo, Finland; ^2^Department of Psychology, The Hebrew University of Jerusalem, Jerusalem, Israel; ^3^International Laboratory of Social Neurobiology, Institute for Cognitive Neuroscience, Higher School of Economics, Moscow, Russia; ^4^MAGICS-Aalto, Aalto University, Espoo, Finland; ^5^Baruch Ivcher School of Psychology, Interdisciplinary Center Herzliya, Herzliya, Israel

**Keywords:** empathy, neural oscillations, alpha rhythm, neurophenomenolgy, pain empathy, magnetoencephalagraphy, social neuroscience

## Abstract

Empathy is often split into an affective facet for embodied simulation or sometimes sensorial processing, and a cognitive facet for mentalizing and perspective-taking. However, a recent neurophenomenological framework proposes a graded view on empathy (i.e., “*Graded Empathy*”) that extends this dichotomy and considers multiple levels while integrating complex neural patterns and representations of subjective experience. In the current magnetoencephalography study, we conducted a multidimensional investigation of neural oscillatory modulations and their cortical sources in 44 subjects while observing stimuli that convey vicarious pain (vs no-pain) in a broad time window and frequency range to explore rich neural representations of pain empathy. Furthermore, we collected participants’ subjective-experience of sensitivity to vicarious pain, as well as their self-reported trait levels of affective and cognitive empathy to examine the possible associations between neural mechanisms and subjective experiences and reports. While extending previous electrophysiological studies that mainly focused on alpha suppression, we found here four significant power modulation patterns corresponding to multiple facets of empathy: an early central (peaking in the paracentral sulcus) alpha (6–11 Hz) suppression pattern plausibly reflecting sensory processing, two early beta (15–23 Hz) suppression patterns in the mid-cingulate cortex (plausibly reflecting the affective component) and in the precuneus (plausibly reflecting the cognitive component), and a late anterior (peaking in the orbitofrontal cortex) alpha-beta (11–19 Hz) enhancement pattern (plausibly reflecting cognitive-control inhibitory response). Interestingly, the latter measure was negatively correlated with the subjective sensitivity to vicarious pain, thereby possibly revealing a novel inhibitory neural mechanism determining the subjective sensitivity to vicarious pain. Altogether, these multilevel findings cannot be accommodated by the dichotomous model of empathy (i.e., affective-cognitive), and provide empirical support to the *Graded Empathy* neurophenomenological framework. Furthermore, this work emphasizes the importance of examining multiple neural rhythms, their cortical generators, and reports of subjective-experience in the aim of elucidating the complex nature of empathy.

## Introduction

Feeling other individuals’ pain and suffering, known as pain empathy, facilitates human social interactions. Empathy has received great attention in the past two decades and neuroscientific studies have demonstrated the involvement of several different underlying brain networks suggesting two subsystems for empathy: (a) an emotional component involving sensory and affective neural substrates such as the sensorimotor cortex, anterior insula, and anterior and middle cingulate cortex (ACC and MCC); and (b) a higher-order cognitive component that reflects vicarious understanding and theory of mind (TOM) involving regions such as the precuneus/posterior cingulate cortex, temporoparietal junction, and prefrontal cortex (Jackson et al., 2005; Cheng et al., 2008; Shamay-Tsoory et al., 2009; Lamm et al., 2011; Bernhardt and Singer, 2021; Zhou and Han, 2021). Furthermore, a number of these brain regions were examined by transcranial magnetic stimulation revealing their causal role in pain empathy and empathic behavior (Avenanti et al., 2005; Gallo et al., 2018; Yang et al., 2018; Zeugin et al., 2020). So far, electroencephalography (EEG) and magnetoencephalography (MEG) studies on empathy for vicarious pain mainly reported modulation of central-parietal-sensory alpha frequency band (7–13 Hz) oscillations (mu rhythm) suggesting that this phenomenon reflects embodied simulation, in line with the prominent affective (i.e., embodied simulation)-cognitive (i.e., mentalizing) empathy model (Perry et al., 2010; Whitmarsh et al., 2011; Woodruff et al., 2011; Chen et al., 2012; Hoenen et al., 2015; Motoyama et al., 2017; Rieèanskı and Lamm, 2019). The rationale behind the phenomenon of pain empathy mainly relies on the resonance/mirroring phenomenon during which the observation of vicarious pain elicits painful sensations in the observer (Osborn and Derbyshire, 2010). Hence neuroscientists typically dichotomize and argue that pain empathy relies on sensory/embodied-simulation (Lamm et al., 2011) while the cognitive facet of empathy is missing except during explicit instructions for mentalization (Lamm et al., 2007; Fan and Han, 2008). However, a recent neurophenomenological framework challenges the affective-cognitive dichotomy and suggests not to search for a single set of brain areas for a certain type of empathy but instead to examine the complex multi-rhythmicity in the cortex together with the individual’s subjective experiences such as social dynamics, lived encounters, and feedbacks (Levy and Bader, 2020). They asserted that integrating subjective experiences with multi-faceted neuroscientific findings provides a more accurate and comprehensive outlook to describe the experience of empathy.

Thus far, the studies that looked into neural rhythms underlying empathy mainly reported the involvement of the alpha rhythm (Perry et al., 2010; Whitmarsh et al., 2011; Woodruff et al., 2011; Chen et al., 2012; Hoenen et al., 2015; Motoyama et al., 2017; Rieèanskı and Lamm, 2019). Alpha-band activity is involved in numerous emotional and cognitive processes (Klimesch et al., 2007; Hanslmayr et al., 2012; Bauer et al., 2014; Frey et al., 2015; Sadaghiani and Kleinschmidt, 2016; Schubring and Schupp, 2021), and in particular, it has a unique dual functionality: a cortical inhibitory control role reflected by an increase in alpha band power (i.e., enhancement) as well as an active role “gating by inhibition” (Jensen and Mazaheri, 2010). Accordingly, alpha power suppression is thought to reflect release from inhibition in the brain (Pfurtscheller and Lopes da Silva, 1999; Mazaheri et al., 2009; Haegens et al., 2010; Jensen and Mazaheri, 2010). In addition to these multiple studies on the involvement of alpha suppression vs enhancement in cognition, a recent series of studies point to its involvement in affective processing of vicarious pain (Whitmarsh et al., 2011; Rieèanskı et al., 2015; Levy et al., 2018) and distress (Levy et al., 2016, 2019a,b, 2019c; Pratt et al., 2016) as well as inhibitory control in response to negative emotional stimuli (Schubring and Schupp, 2021). Furthermore, there are other aspects of alpha rhythmicity which deserve attention: timing (e.g., early vs late) and phase-locking (e.g., induced vs evoked activity), just like other studies on working memory (Deiber et al., 2007) and emotion (Schubring and Schupp, 2021). In particular, while few studies examined induced neural response during empathy (Levy et al., 2016, 2018), induced activity reflects integrative functions, and not only externally-evoked processes and is therefore crucial not to overlook (Tallon-Baudry and Bertrand, 1999). Hence, the examination of the alpha rhythm during the process of empathy should not relate to alpha as a uni-dimensional phenomenon, but rather to multiple features such as suppression vs enhancement, timing and phase-locking.

Despite the almost exclusive focus on the role of the alpha rhythm in empathy, a few studies reported the involvement of the beta rhythm. However, none of these studies inspected the sources of beta activity in the brain and expounded the role of beta oscillations in empathetic responses (Whitmarsh et al., 2011; Rieèanskı et al., 2015; Levy et al., 2018). More broadly, the functional role of beta-band oscillations in cognitive and perceptual processing has been reviewed (Engel and Fries, 2010; Bressler and Richter, 2015), and it has been proposed that this rhythm is associated with the maintenance of the current processing or so-called “*status quo.*” In other words, the modulation in beta-band power is thought to reflect the involvement in the top-down cognitive processing applied by an unexpected external stimulus. Hence, these converging lines of research emphasize the need for further investigation of the role that beta oscillations play during the experience of empathy and distinguishing its functional contribution from that of the alpha rhythm.

Notwithstanding the importance of inspecting complex neural rhythmicity, another crucial aspect is the subjective experience of empathy, or in other words, its phenomenological representation (Zahavi, 2012). By focusing on the subjective experience of empathy, phenomenological studies show that empathy is not dichotomous but rather a graded process (Stein, 1989; Fuchs, 2017). Recently, Grice-Jackson and colleagues demonstrated that the affective-cognitive dichotomy cannot straightforwardly accommodate neuroimaging representations of pain empathy that incorporate also its subjective representations (Grice-Jackson et al.,2017a,b). Specifically, the authors implemented a task [vicarious pain questionnaire (VPQ)] that presented vignettes of individuals in painful situations, and it inquired about the graded level of the subjective experience of self-pain while perceiving vicarious pain.

The main goal of the current study is to test whether pain empathy can be represented as a graded phenomenon, inspired by the *Graded Empathy* framework. Specifically, we test whether empathy can extend beyond the dichotomous view of embodied-simulation vs cognitive facets, and beyond the exclusive focus on distinct neural substrates (in neuroimaging studies) or on the alpha rhythm (in electrophysiological studies). Hence, we examine the multiple rhythmic aspects of MEG signal during pain empathy by inspecting a broad frequency band, long time window, and induced activity. Moreover, we investigate the cortical generators of these brain oscillations (Baillet, 2017; Gross, 2019) to facilitate the interpretation of their functional role in pain empathy. We hypothesize that the multidimensional examination of neural patterns will reveal a multifaceted, rather than dichotomous, neural representation of pain empathy including sensory, affective, cognitive, bottom-up and top-down components. Finally, we further examine the nature of the potential link between these neural representations and reports of subjective-experience and cognitive-affective traits. Specifically, we collect reports on subjective-experience during pain empathy (Grice-Jackson et al., 2017a) and on affective-cognitive traits (IRI; Davis, 1983), and test two predictions: that the brain-experience correspondence is either graded (i.e., as a function of subjective-experience rating) or dichotomous (i.e., functionally divided by affective-cognitive traits), thereby providing an additional examination of the graded vs the dichotomous frameworks.

## Materials and Methods

### Participants

Forty-four healthy adult subjects (19 females, mean age ± SD = 25.7 ± 3.94) were recruited for this study. MEG compatibility and history of psychiatric and neurological disorders were checked before the recruitments. All instructions were presented in the participant’s mother tongue and subjects were given compensation for participation in this study. The study was approved by the IDC Herzliya ethics committee, and the consent form was signed by all participants.

### Experimental Design

#### MEG Session

Subjects lay in supine position inside the MEG scanner while facing a screen projecting the stimuli at a viewing distance of approximately 55 cm. The stimuli and design were similar to our previous experiments (Levy et al., 2016, 2018, 2019b; Pratt et al., 2016). Well validated 96 color pictures of limbs (48 in pain and 48 in no-pain conditions) appeared in uniform size (300 × 225 pixels) at the center of a gray background on a 20-inch monitor. We used the pain (P) condition to elicit empathy for pain and the no-pain (NP) to control other parameters induced by the visual stimuli. Subjects were trained to remain relaxed and watch the presented stimuli. Stimuli were randomly presented for 1 s with inter-stimulus intervals of 2.5–3.3 s of fixation crosshair. To keep and assess the subject’s attention, we created twirl filler trials using a short twisted movement in new stimuli (Photoshop, Adobe Systems Inc.) and randomly presented them to the participants. Subjects were trained to press the response button when detecting the twirl stimuli. The filler trials were not analyzed. The experiment was programmed and operated by E-Prime^®^ software (Psychology Software Tools Incorporated).

#### Self-Rating Session

To evaluate the self-reported (trait) and subjective-experience (state) empathy, before the neuroimaging measurements, subjects were asked self-report the following tasks: First, they rated their levels of “empathic concern” and “perspective taking” subscales of the IRI questionnaire (Davis, 1983) to assess participant’s empathy traits. Second, participants’ subjective experience of sensitivity to vicarious pain was evaluated with VPQ (Grice-Jackson et al., 2017a), a qualitative method using 14 painful videos to measure pain perception. Participants rated the level of discomfort they felt by watching each one of the fourteen vignettes. We then computed the average score for all fourteen rating scores.

### Data Acquisition and Preprocessing

Inside a magnetically shielded room, participants’ brain activity was recorded with a sampling rate of 1,017 Hz (online 1–400 Hz band-pass filter) using a whole-head MEG with a 248-channel magnetometer array (4-D Neuroimaging, Magnes^®^ 3600 WH). Five coils were attached to the subjects’ scalp to record head position relative to the sensor. Environmental noise was canceled by placing reference coils approximately 30 cm above the subject’s head and orienting them by the *x*, *y*, and *z* axes. All the data preprocessing and analysis were performed using MATLAB 2014b (MathWorks) and the FieldTrip software toolbox. We removed eye movement, eye blink, and heart artifacts using independent component analysis and visually checked and rejected any remaining bad trials. We band-pass filtered in the 1–150 Hz, and analyzed data of 2,500 ms epochs including a baseline period of 450 ms.

### Sensor and Source Analysis

#### Sensor

A Hanning taper was applied to each epoch of the 248-sensor data To evaluate Time-Frequency Representations (TFRs) of alpha and beta power for each trial and to compute the Fast Fourier Transform (FFT) for short sliding time windows of 0.5 s (spectral resolution of 2 Hz) in the 1–150 Hz frequency range. Data were analyzed in alignment with the onset of the stimuli and averaged power across tapers was computed. A Hanning taper, applied to each epoch yielded the FFT for short sliding time windows of 0.5 s in the 1–40 Hz frequency range, resulting in a spectral resolution of 2 Hz. To probe gamma-frequency power (40–150 Hz), five Slepian multitapers were applied using a fixed window length of 0.2 s, resulting in a frequency smoothing of 15 Hz. Evoked responses were subtracted from the induced activity as required while studying top-down cognitive tasks in the brain. Eventually, TFRs for the statistically significant contrast two conditions (P and NP) were calculated.

#### Source

To localize the source activity, we used SPM8 (Wellcome Department of Imaging Neuroscience, University College London, www.fil.ion.ucl.ac.uk) to manually digitize the head shape (Polhemus FASTRAK^®^ digitizer), and build a single shell brain model based on an MNI adult template brain. Then, we modified the model for each subject to fit their digitized head shape. To perform group analysis, each subject’s brain volume was divided into a regular 1 cm grid. Then a beamformer was applied to reconstruct a spatial filter (Gross et al., 2001) for each grid position to pass activity from the single location of interest in the statistically significant sensor time-frequency windows and block the activity of all other locations.

### Statistical Analysis

To do statistical group analysis, we used a non-parametric statistical approach (Maris, 2007). First, the *t*-value of contrast between P and NP conditions was calculated per subject, channel, frequency, and time and then, the test statistic was defined by pooling the *t*-values over all subjects. We permuted the original conditions in each subject by randomly multiplying each subject’s *t*-value by 1 or –1 and summing over subjects to evaluate time-frequency clusters with a significant effect. This cluster-based randomization procedure was repeated 1,000 times to produce a randomization distribution. Finally, significance thresholds for a two-sided test were corrected by multiple comparisons method using maximum/minimum clusters, and Monte Carlo significance probability (*P*-value; Maris, 2007) was evaluated by computing the proportion of values that exceed the test statistic in the randomization distribution.

## Results

### MEG Sensor-Level Results

**FIGURE 1:**
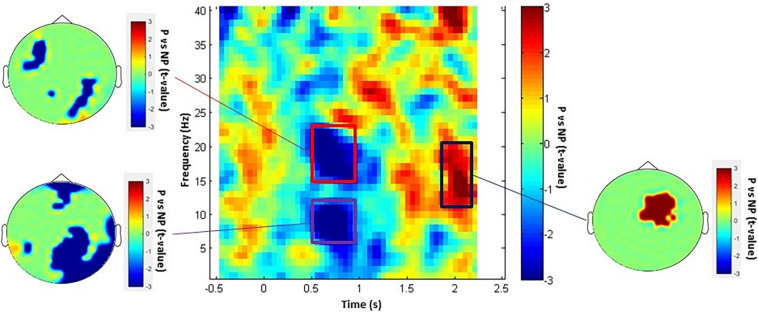
P vs NP TFR and topographical maps. The TFRs include time window of –0.5 to 2.5 s (averaged over all channels) and topography of each statistically significant time-frequency window. The rectangular insets represent time-frequency windows of activity that were statistically significant (P_cluster–cor_ < 0.05).

We investigated the neural effect of empathy while participants were watching painful (P) and non-painful (NP) pictures inside the MEG scanner. We probed the neural rhythmicity modulation at the whole sensor-array level in the time window of 0–2.5 s and 1–150 Hz frequency range. As represented in Figure 1, the statistical time-frequency contrast map averaged across sensors in the 1–40 Hz range reveals three significant (P*_cluster–cor_* < 0.05) time-frequency patterns in response to observing P vs NP. Significant alpha (6–12 Hz) and beta (15–23 Hz) suppression pattern was exhibited in the time window of approximately 500–1,000 ms and a surprising significant alpha/low-beta (11–19 Hz) enhancement was detected 1,800–2,300 ms after stimulus onset. Topographies of *t*-values averaged across each significant time and frequency bins illustrate the most modulated brain regions. Topography of alpha (6–12 Hz) changes in the time window of 500–1,000 ms indicates power decrease over central-posterior regions, whereas beta (15–23 Hz) was suppressed in various non-localized sensors. Further, the late enhancement pattern in high-alpha/low-beta (11–19 Hz) was observed under antero-central sensors. Finally, TFR in the 40–150 Hz range revealed no-significant (P_cluster–cor_ > 0.71) differences between P and NP.

### MEG Source-Level Results

**FIGURE 2:**
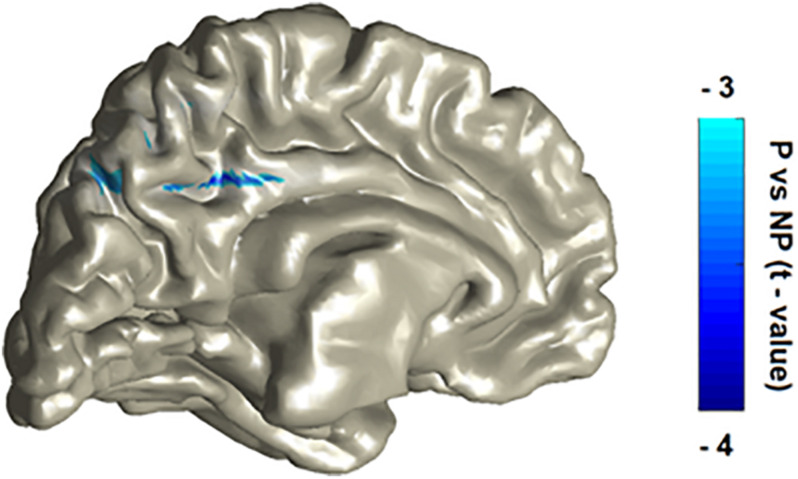
P vs NP statistical contrast of the source localization of the beta suppression effect. The localization procedure reveals two main peaks, in the cingulate cortex and in the precuneus. The patterns were laid over on MNI template with a color bar representing masked and peak statistical activity (P_cluster–cor_ < 0.05).

To probe the exact source of modifications, we conducted source localization on each one of the three significant time-frequency windows selected during sensor analysis. One participant was excluded from source analysis due to excessive head movement (deviation of more than 3 cm). First, in the early alpha suppression window, we found a statistical tendency (P_cluster–cor_ = 0.09) with a peak source in the paracentral sulcus, in line with the topoplot result and replicating the typical central-parietal-sensory alpha suppression response in the literature. Second, the concurrent beta suppression was found to emanate from two significant (P_cluster–cor_ < 0.05) sources: the middle cingulate cortex (i.e., a typical simulation-affective region) as well as the precuneus (i.e., a typical mentalizing-cognitive region). Third, in the late alpha-beta enhancement window, we found a statistical tendency (P_cluster–cor_ = 0.09) with a peak source in the orbito-frontal cortex (OFC) in line with the topoplot result and congruent to two recent EEG experiments (Schubring and Schupp, 2021). Figure 2 illustrates the robustly significant source maps (P_cluster–cor_ < 0.05), that is, the beta sources.

### Self-Reported Results

**FIGURE 3:**
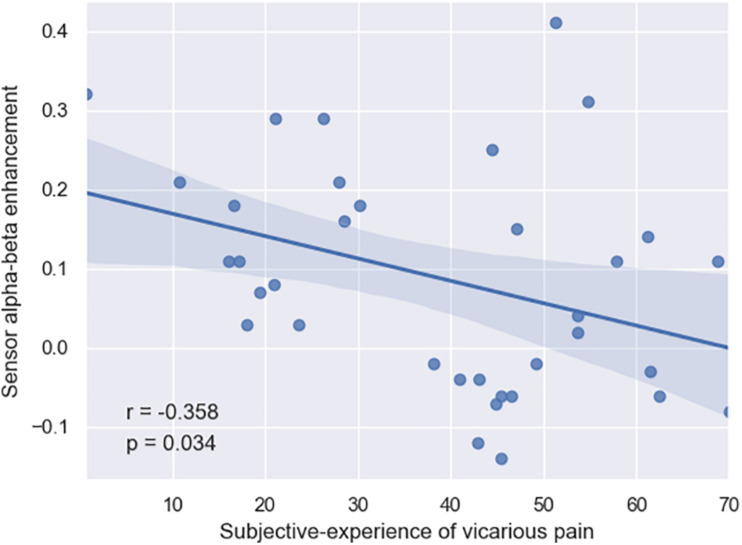
Negative correlation between the subjective experience of subjects and their late alpha-beta power enhancement in the brain (*r* = –0.358, *p* = 0.034).

Finally, we conducted Spearman correlations between the (i) three neural patterns and the (ii) self-reports of subjective-experience and affective-cognitive traits. Overall, none of the neural patterns significantly (*p* > 0.18) correlated with the affective-cognitive traits. By contrast, whereas the suppression patterns did not significantly (*p* > 0.24) correlate with subjective-experience, the enhancement pattern did (*r* = –0.358; *p* = 0.03), thereby suggesting that so that more enhancement in the late alpha-beta power (i.e., inhibitory control) is associated with less sensitivity to vicarious pain (Figure 3).

## Discussion

Empathy is a complex social ability in the human species with multiple facets, ranging from low-level sensory and affective aspects to high-level cognitive aspects that involve top-down processes in the brain and even further aspects based on the social circumstances indicated by phenomenological analysis (Levy and Bader, 2020). The present study aimed to move beyond the dualistic affective-cognitive representation of empathy by exploiting the richness of data collected in MEG in accordance to recent multilevel models on empathy (Schurz et al., 2021; Weisz and Cikara, 2021), and in particular *Graded Empathy* framework that connects neural rhythms and subjective experience. Despite the simplicity and artificial nature of the task employed here, we investigated multiple dimensions of rhythmic neural patterns during empathy for vicarious pain. We identified early and late, suppressions and enhancements of multiple rhythms and their cortical generators, and explored their associations with self-reports of subjective-experience and trait empathy.

Previous electrophysiological studies (EEG and MEG) on pain empathy typically focused on the basic aspects of empathy and repeatedly showed suppression in alpha power in central-parietal regions in a few hundreds of milliseconds after stimulus onset (Cheng et al., 2008; Perry et al., 2010; Whitmarsh et al., 2011; Woodruff et al., 2011; Chen et al., 2012; Hoenen et al., 2015; Motoyama et al., 2017; Rieèanskı and Lamm, 2019). For instance, Whitmarsh et al. (2011) who detected a significant alpha suppression in the sensory cortices while observing pain (compared to no-pain) pictures, argued that based on the “gating to inhibition” hypothesis, this decrease in alpha power has a disinhibitory role in sensory cortices for empathetic responses. In the current MEG study, we replicated their results and similarly observed an alpha suppression pattern in the sensory region (with peak source at paracentral sulcus, though the cortical localization effect yielded a statistical trend) in an earlier time window (probably due to onset latency) which represents gating sensory information to the sensorimotor cortex in response to observing painful stimuli. However, in addition to the sensory alpha oscillation, we extend the current literature by detecting several other patterns reflecting other facets of empathy: two distinct cortical generators of a concurrent beta suppression pattern, and a late frontal alpha-beta enhancement pattern. We further elaborate below on these new neural representations of empathy.

Although the functional role of beta power oscillation is not well-understood, recent studies demonstrated the role of beta oscillatory activity in processing higher-order information in the brain, namely in endogenous top-down processing of cognitive and perceptual tasks (Engel and Fries, 2010). For instance, studies on working memory indicated beta-band modulation during matching stimulus detection (Tallon-Baudry et al., 2001; Deiber et al., 2007). Additionally, some studies denoted the relation of beta activity to the behavioral context of top-down signals (Engel and Fries, 2010; Bressler and Richter, 2015; Friston et al., 2015). By conducting source localization, we determined the exact location of beta rhythm changes: one of the beta suppression patterns was estimated to be generated by sources in the MCC. The functional magnetic resonance imaging (fMRI) literature on pain empathy highlights the cingulate cortex as a core part of the network involved in self and others’ pain processing (Lamm et al., 2011; Yesudas and Lee, 2015) and vicarious unpleasantness (Ionta et al., 2020). Evidence demonstrated the role of ACC and MCC in shared affective mirroring of the unpleasantness of the observed pain so that similar neurons fire during self-experiencing of pain and observation of pain in other individuals. Therefore, this significant beta-band suppression in MCC in response to vicarious pain most probably reflects the MCC activation representing the affective aspect of empathy.

The other beta suppression was estimated to emanate from the precuneus region. The role of the precuneus in processing multiple cognitive functions such as perspective-taking, mentalizing and TOM was demonstrated previously (Farrow et al., 2001; Cavanna and Trimble, 2006; Arora et al., 2017). Functional neuroimaging studies on empathy highlighted the precuneus as a major part of the network involved in the cognitive facet of empathy (Cavanna and Trimble, 2006; Morelli et al., 2014; Fauchon et al., 2019). We speculate that the latter beta suppression in the precuneus region indicates the cognitive component of empathy, including mentalizing, TOM, and perspective-taking. These beta oscillatory findings suggest several facets of empathy – not only sensorial but also affective and cognitive Although to date very little research has examined the cortical generators of the beta rhythm during pain empathy, a previous study showed very similar activation patterns in the parietal cortex, and even in the MCC [noteworthy, the latter was found in a group of 80 adolescents (Levy et al., 2018)]. It is important, however, that more studies in the future replicate these findings and elucidate the functional role of beta oscillation during the experience of empathy.

Furthermore, we interestingly discovered a late increase in alpha-beta power plausibly originating from the OFC (noteworthy, the cortical localization effect yielded a statistical trend). Based on former evidence, there is an association between alpha power enhancement and inhibition in the task-irrelevant brain regions: Many MEG and EEG studies on motor functioning, attention, and memory reported the increase in alpha activity as a marker of active inhibition of sensory information in a particular brain area (Mazaheri et al., 2009; Haegens et al., 2010; Uusberg et al., 2013). Besides, other lines of research indicated the role of OFC in the regulation of human emotion and social behavior by inhibiting irrelevant or uncomfortable stimuli (e.g., negative and painful sensations; Ochsner and Gross, 2005; Hooker and Knight, 2006; Hartikainen et al., 2012; Bryden and Roesch, 2015). More specifically, OFC automatically disrupts and filters negative affective information coming through the brain from the internal and external environment (Hooker and Knight, 2006). Considering the active inhibitory role of alpha enhancement as well as OFC regulatory role, we suggest that the late OFC alpha-beta power enhancement detected in the current empathy study reflects a top-down inhibitory control mechanism in perceiving painful stimuli to regulate emotion and social behavior. Our findings are partially in line with a recent article examining three different EEG studies on negative and positive high arousal emotions (first study: erotic vs neutral; second study: mutilation vs neutral; third study: erotic vs mutilation; Schubring and Schupp, 2021). Schubring and Schupp reported an early alpha/low-beta (10–16 Hz) suppression in response to observing mutilation pictures over the central sensors, showing activation at the sensory area as well as a late alpha/low-beta (10–20 Hz) enhancement over anterior and posterior EEG sensors in response to observing negative but not positive high arousal stimuli, representing functional inhibitions to negative stimuli. Despite the differences in experimental paradigms and electrophysiological methodologies, the present enhancement finding is very similar to that reported by Schubring and Schupp. Our use of MEG enabled us to further explore the cortical generator of this effect and add knowledge and understanding about this top-down mechanism involved in empathy.

Moreover, even though we did not detect any significant correlation between neural patterns and affective-cognitive traits, by integrating subjects’ life experiences, we found a significant negative correlation of the detected late enhancement of alpha-beta power with subjective sensitivity to others’ pain suggesting that the late neural inhibition may act as a mechanism for inhibiting sensitivity to vicarious pain. This finding indicated that the dichotomous affective-cognitive view does not straightforwardly accommodate human lived experiences and empathic encounters, and rather supports the *Graded Empathy* framework (Levy and Bader, 2020). The results suggest that individuals with greater late alpha-beta enhancement have lower sensitivity to vicarious pain, whereas people with high sensitivity to vicarious pain have less inhibitory control in their brain (Weisz and Cikara, 2021), thereby plausibly enabling them to empathize with others’ pain. This is in agreement with former studies on the relation of individual’s experiences through lifespan development with their functional architecture for the cognitive control of emotion (Ochsner and Gross, 2005). Accordingly, up-regulation or down-regulation of emotion by the top-down cognitive control directs one’s empathic response toward others as has been suggested in early and recent accounts on empathy (Decety and Lamm, 2006; Weisz and Cikara, 2021). Future studies should further elucidate this interesting, plausibly top-down driven, pattern by conducting connectivity analyses that would explore information trafficking across networks, and in-depth phenomenological interviews that would add the phenomenological dimension of this cognitive control phenomenon.

A recent developmental study on pain empathy denoted gradual shifts of brain oscillatory activities from primary uni-rhythm sensory activity in childhood to higher-order multi-rhythmic oscillations in adulthood (Levy et al., 2018). They found significant alpha and beta power suppression as well as gamma power enhancement particularly in adults with an average age of approximately 41 years old. They interpreted visceromotor gamma activity as a neural marker of empathy development from self-based to other-focused representing a deeper understanding of others. In the current study, even though we observed alpha and beta suppression in subjects with an average age of about 26 years old, we did not detect any significant gamma oscillatory activity, suggesting that full-blown empathy maturation may develop at a later age, and not in the mid-twenties.

Finally, although we detected beta power modulations from both affective and cognitive networks, we additionally detected a sensory alpha power suppression pattern (reflecting sensory aspect) and frontal alpha-beta power enhancement pattern (reflecting cognitive control processes), albeit the alpha cortical localization effect yielded a statistical trend. This suggests that there is no dichotomy but a multifaceted representation for pain empathy which can be confirmed by lack of correlations between the neural patterns and affective or cognitive trait empathy reports and the correlation of alpha-beta power enhancement pattern with the subjective experience. Lack of neural correlation with trait empathy reports is in line with the recent discussions in the literature regarding the limitation of IRI trait self-report in measuring all aspects of empathy (DiGirolamo et al., 2019). Yet, it is important to consider that the nature of the painful stimuli category might affect the neural correlation with subjective experiences or lack of correlation with affective or cognitive trait empathy. This can be further investigated by examining an alternative sort of painful stimuli (e.g., emotional painful stimuli). Besides, it is worthwhile to note that interpreting the functional role of each oscillatory activity in this empathy study is based on the previous literature, and using fMRI alongside MEG in future studies can provide further insight into the functional role of each of these brain oscillations. In terms of phenomenological evaluation, although we assessed the subjective experience of vicarious pain, thereby emulating phenomenological assessment, future studies need to conduct in-depth interviews that would more deeply explore participants’ thoughts, emotions, beliefs and experiences (Bockelman et al., 2013). Notwithstanding these limitations, the current study points out a new approach and empirical evidence that empathy extends beyond the affective-cognitive dichotomy while triggering a graded cascade of rhythmic representations of simulation, affect, mentalization, cognitive-control and subjective-experience.

## Data Availability Statement

The raw data supporting the conclusions of this article will be made available by the authors upon reasonable request, pending institutional ethical policies.

## Ethics Statement

The studies involving human participants were reviewed and approved by IDC Herzliya Ethics Committee. The patients/participants provided their written informed consent to participate in this study.

## Author Contributions

JL and EA contributed to conception and design of the study. EA collected data. NZ, EA, and AK analyzed the data. NZ and JL wrote the manuscript. JL, MS, and IJ contributed funding to support the study. All authors contributed to manuscript revision, read, and approved the submitted version.

## Conflict of Interest

The authors declare that the research was conducted in the absence of any commercial or financial relationships that could be construed as a potential conflict of interest.
